# Treatment of leukemia antigen-loss relapses occurring after CD19-targeted immunotherapies by combination of anti-CD123 and anti-CD19 chimeric antigen receptor T cells

**DOI:** 10.1186/2051-1426-3-S2-O5

**Published:** 2015-11-04

**Authors:** Marco Ruella, David M Barrett, Saad S Kenderian, Olga Shestova, Ted J Hofmann, John Scholler, Simon F Lacey, Jan J Melenhorst, Farzana Nazimuddin, Jessica Perazzelli, David A Christian, Christopher A Hunter, David L Porter, Carl H June, Stephan A Grupp, Saar Gill

**Affiliations:** 1 Center for Cellular Immunotherapies, Perelman School of Medicine at the University of Pennsylvania, Philadelphia, PA, USA; 2 Department of Pediatrics, Perelman School of Medicine at the University of Pennsylvania, Philadelphia, PA, USA; 3 Department of Pathobiology, School of Veterinary Medicine at the University of Pennsylvania, Philadelphia, PA, USA; 4 Division of Hematology-Oncology, Department of Medicine, Perelman School of Medicine at the University of Pennsylvania, Philadelphia, PA, USA

## Introduction

Anti-CD19 chimeric antigen receptor T cells (CART19) and bi-specific anti-CD19/CD3 antibodies (blinatumomab) are generating unprecedented complete responses in relapsing/refractory B-cell acute lymphoblastic leukemia (r/r B-ALL). However, a subset of patients still relapse and about 30-50% of these relapses are characterized by the loss of detectable CD19 [1-3]. The interleukin-3 receptor alpha, or CD123, was shown to be expressed in several hematologic neoplasms, including acute myeloid leukemia and more recently also B-ALL. The goal of this study was to pre-clinically evaluate the impact of targeting both CD19 and CD123 with chimeric antigen receptor T cells for the treatment and prevention of CD19-negative relapses occurring after CD19-directed therapies [4,5].

## Results

**Figure 1:**
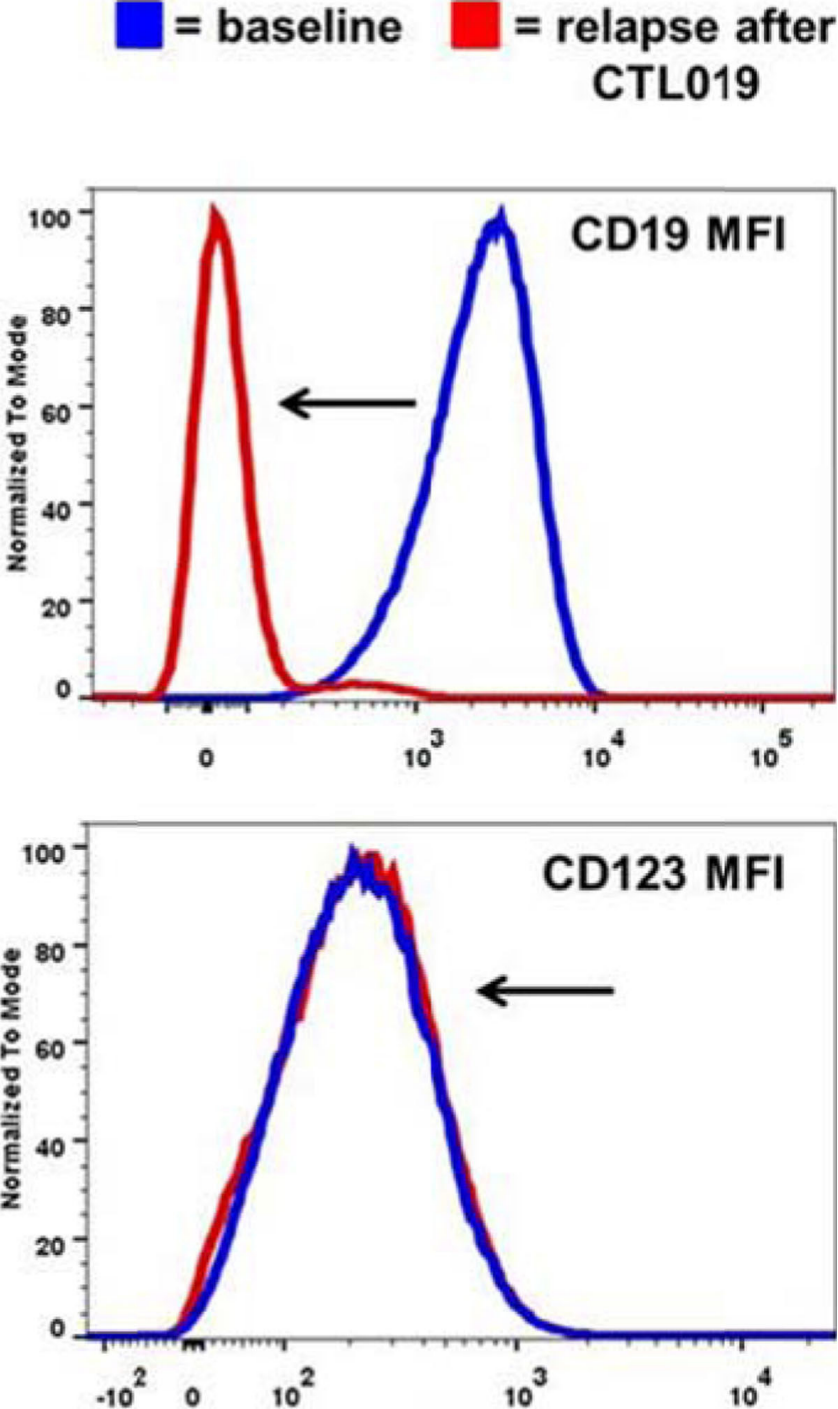


**Figure 2:**
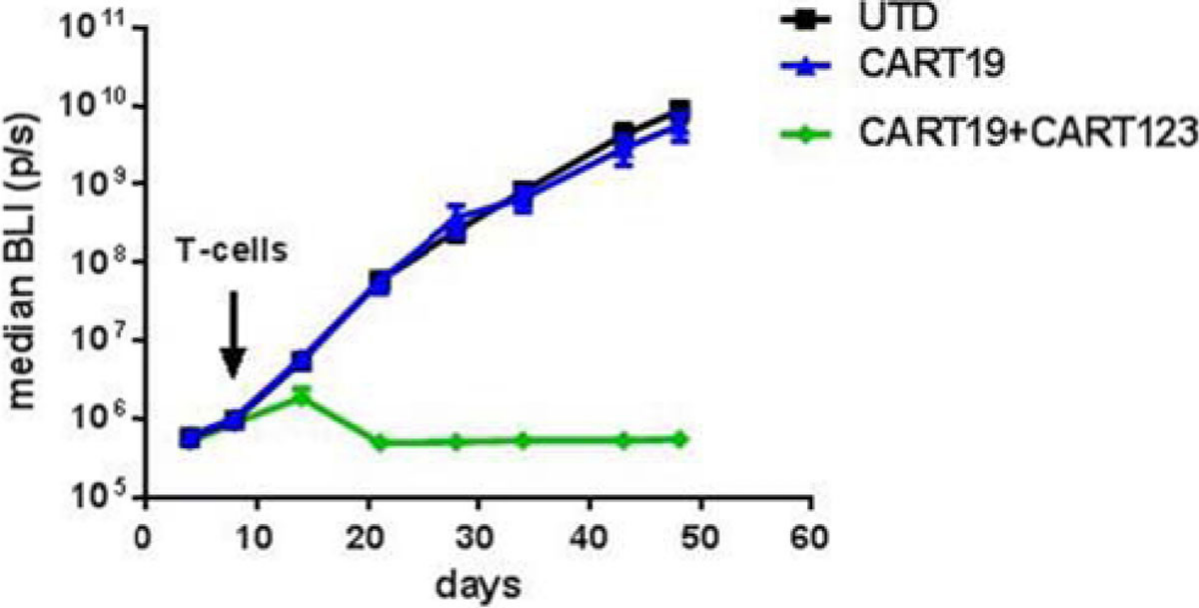


CD123 expression was analyzed by flow cytometry in 36 r/r B-ALL samples: CD123 was highly expressed (81.75%, range: 5.10-99.60), representing a promising candidate for targeted therapy in B-ALL. Moreover, CD123 was also found to be expressed in the putative leukemia stem cells, identified as CD34-pos CD38-neg. The expression of CD123 was detected in all (n=6) CD19-negative B-ALL blasts analyzed after relapse from CART19 treatment (representative case in Figure 1). Therefore, we generated anti-CD123 chimeric antigen receptor T cells co-stimulated with 4-1-BB using a lentiviral vector (CART123) [5]. We then evaluated the CART123 anti-leukemia efficacy both *in vitro* and *in vivo* against primary B-ALL blasts and the cell line NALM-6. CART123 showed intense anti-leukemia activity, as defined by specific CD107a degranulation, cytokine production, cytotoxicity and proliferation, not statistically different from that of CART19. In order to test the role of CART123 to target CD19- negative relapses we developed a novel *in vivo* model, engrafting immunodeficient NSG mice with blasts from a patient relapsing with CD19-negative disease after CART19 treatment. At day 14 mice were randomized to receive control T cells (UTD), CART19, or CART123 in combination with CART19. CART19 and control T cell treated mice showed no anti-tumor activity, while CART123+CART19 led to complete eradication of the disease and long-term survival (Figure 2).

## Conclusions

Here we demonstrate that CD123 is expressed in CD19-negative B-ALL relapses occurring after CD19-directed therapies, and that combining CART123 cells with CART19 cells is an effective therapy for the treatment and prevention of antigen-loss relapses in B-ALL murine xenografts.
